# Automatic Planning of Whole Breast Radiation Therapy Using Machine Learning Models

**DOI:** 10.3389/fonc.2019.00750

**Published:** 2019-08-07

**Authors:** Yang Sheng, Taoran Li, Sua Yoo, Fang-Fang Yin, Rachel Blitzblau, Janet K. Horton, Yaorong Ge, Q. Jackie Wu

**Affiliations:** ^1^Department of Radiation Oncology, Duke University Medical Center, Durham, NC, United States; ^2^Medical Physics Graduate Program, Duke University Medical Center, Durham, NC, United States; ^3^Department of Radiation Oncology, University of Pennsylvania, Philadelphia, PA, United States; ^4^Department of Software and Information Systems, University of North Carolina, Charlotte, NC, United States

**Keywords:** whole breast radiation therapy, breast cancer, machine learning, auto planning, random forest, electronic compensation

## Abstract

**Purpose:** To develop an automatic treatment planning system for whole breast radiation therapy (WBRT) based on two intensity-modulated tangential fields, enabling near-real-time planning.

**Methods and Materials:** A total of 40 WBRT plans from a single institution were included in this study under IRB approval. Twenty WBRT plans, 10 with single energy (SE, 6MV) and 10 with mixed energy (ME, 6/15MV), were randomly selected as training dataset to develop the methodology for automatic planning. The rest 10 SE cases and 10 ME cases served as validation. The auto-planning process consists of three steps. First, an energy prediction model was developed to automate energy selection. This model establishes an anatomy-energy relationship based on principle component analysis (PCA) of the gray level histograms from training cases' digitally reconstructed radiographs (DRRs). Second, a random forest (RF) model generates an initial fluence map using the selected energies. Third, the balance of overall dose contribution throughout the breast tissue is realized by automatically selecting anchor points and applying centrality correction. The proposed method was tested on the validation dataset. Non-parametric equivalence test was performed for plan quality metrics using one-sided Wilcoxon Signed-Rank test.

**Results:** For validation, the auto-planning system suggested same energy choices as clinical-plans in 19 out of 20 cases. The mean (standard deviation, SD) of percent target volume covered by 100% prescription dose was 82.5% (4.2%) for auto-plans, and 79.3% (4.8%) for clinical-plans (*p* > 0.999). Mean (SD) volume receiving 105% Rx were 95.2 cc (90.7 cc) for auto-plans and 83.9 cc (87.2 cc) for clinical-plans (*p* = 0.108). Optimization time for auto-plan was <20 s while clinical manual planning takes between 30 min and 4 h.

**Conclusions:** We developed an automatic treatment planning system that generates WBRT plans with optimal energy selection, clinically comparable plan quality, and significant reduction in planning time, allowing for near-real-time planning.

## Introduction

Breast cancer is the most common non-cutaneous cancer type among females. There are 268,670 estimated new female breast cancer cases in 2018 (1) with additional yearly estimated breast ductal carcinoma *in situ* (DCIS) occurrence of 60,290 (2). Depending on the stage of the cancer, lumpectomy or mastectomy, radiation therapy, chemotherapy and endocrine therapy may all be required. When feasible, many patients opt for a breast conserving surgery and the whole breast radiation therapy (WBRT) is routinely delivered in the post-operative setting to reduce the risk of locoregional recurrence.

WBRT refers to multiple treatment techniques (3–6) including the traditional 3D treatment (7–10) using the physical wedge, the field-in-field (FiF) delivery (11–13), the intensity modulated radiation therapy (IMRT) (14, 15), and the volumetric modulated arc therapy (VMAT) (16–21). Conventionally, a 3D planning technique utilizing the physical wedge was one of the most frequently used treatment methods. The planner determines the wedge specific angle to use and then adjusts the weighting of both beams (the medial and lateral beam) to achieve uniform dose distribution. Since the tunable parameter (wedge angle) for the 3D technique is limited, inhomogeneous dose distribution is often observed within the irradiated volume. The maximum dose for the 3D plan could exceed 110% of the prescription dose, causing a dose hot spot. A large volume hot spot may result in increased acute toxicity for the patient (22). In order to improve dose homogeneity, FiF technique was later implemented. Similar to 3D treatment, FiF uses beam shaping devices [jaws, multi-leaf collimators (MLCs)] to control the delivered fluence. The FiF approach offers the additional benefit of multiple static segments to control the delivered fluence as compared to 3D treatment with 2–4 segments. As a result, dose homogeneity in the irradiated volume is substantially improved. The combination of several segments is essentially equivalent to the use of step-and-shoot IMRT. The FiF treatment planning does not invoke the inverse IMRT optimizer and is therefore a forward planning process.

IMRT with more than two beams and VMAT have also been adopted to deliver the WBRT. Fluence modulation is often favorable for the breast irradiation to compensate for the missing tissue in the beams-eye-view (BEV). However, the inverse planning process makes the treatment planning process less intuitive, since setting the dose volume objectives during optimization can hardly be directly linked to the optimal 3D dose distribution. In addition, the optimization engine may not meet all dose volume goals if the constraints are difficult to achieve. Extra effort (up to several hours) is often needed to improve the homogeneity even if the hot spot volume is small. Computer assistance in whole breast IMRT treatment planning has been studied in recent years. Purdie et al. (23) developed an automatic breast IMRT treatment planning system which adopts a heuristic method. This method mimics the human planning process via setting the beam and optimizing the apertures in a forward planning fashion. VMAT is also applicable for the breast treatment. However, low dose spill to the ipsilateral lung and heart is inevitable for VMAT. Given the high cure rate and long life expectancy for breast patients, limiting normal tissue injuries such as pneumonitis and ischemic heart disease (24) is critical.

As such, WBRT using two classical tangential fields (25) is a preferred treatment option. However, it is not yet the most popular one because improved workflow efficiency is needed due to the large patient volume. Current intensity-modulated methods are more favorable dosimetrically and technically, but require manual painting of the fluence for both beams (the medial and lateral beam) to achieve the uniform dose distribution. In the Eclipse^TM^ (Varian Medical Systems, Palo Alto, CA, USA) treatment planning system (TPS), the planner can start with an initial fluence generated from the “irregular surface compensator” tool. Then the planner modifies the fluence iteratively, and this process has to be re-started when a different beam energy choice or a combination of multiple energies is used. This technique currently requires up to hours of fine-tuning fluence manually, and the plan quality can be highly dependent on the planner's experience.

In this study specifically, we aim to develop an automatic planning system starting with the energy selection, followed by the fluence estimation model using random forest and concluding with a fluence fine tuning module that would enable near-real-time and interactive planning while providing similar plan quality as experienced human planners.

## Materials and Methods

### Materials

A total of 40 institutional review board (IRB) approved WBRT plans from Duke University Medical Center were retrospectively selected for this study. All plans were treated with 200 cGy fractional dose to a total of 25 fractions. Twenty plans, 10 with single energy (SE, 6MV) and 10 with mixed energy (ME, 6/15MV), were randomly selected to establish the optimization parameters of the proposed methodology. The rest 20 plans (10 SE and 10 ME plans) were used as a validation dataset. Among the 20 training cases, 12 are left breast cases. For validation cases, 9 out of 20 are left breast cases. SE plans use two 6MV beams, namely the medial beam and lateral beam, set by the attending physician to include the whole breast and skin flash. For ME plans, two high energy beams (15MV) utilize the same beam setup and beam apertures as two low energy beams (6MV) in the corresponding beam direction. Clinical plans of all 40 cases were manually generated in the Eclipse^TM^ TPS (version 13.6) by planners iteratively painting the fluence and calculating the dose distribution.

### Automatic Whole Breast Radiation Therapy Plan Generation

**Figure 1:**
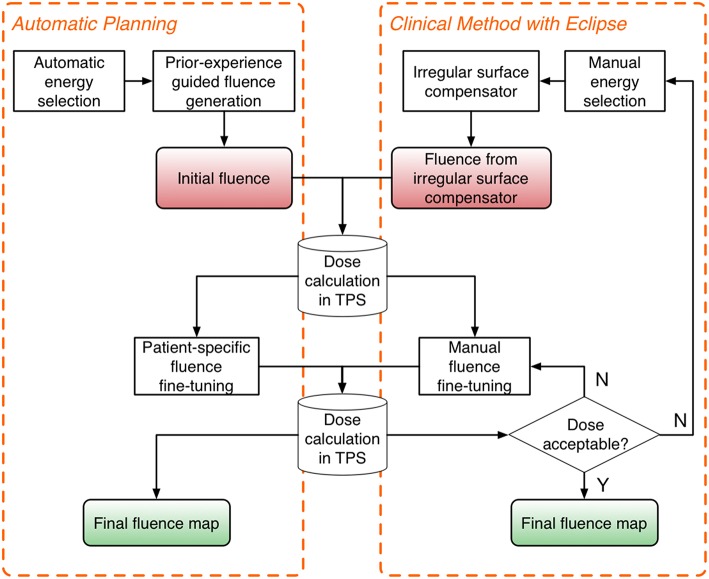
Flowchart of the proposed automatic planning workflow **(Left)** and the current clinical workflow **(Right)**. The automatic planning workflow mimics the workflow of manual planning while providing automation tools to streamline the process.

The automatic planning workflow consists of three major steps: *energy selection, fluence estimation*, and *fluence fine-tuning*. The flowchart of the proposed workflow is shown on the left in Figure 1, and the current clinical workflow is shown on the right for comparison.

#### Digital Reconstructed Radiograph (DRR) Based Energy Selection

For manual planning, planners typically select the beam energy based on the breast size, but may lack confidence in making such decision for moderately-sized breast patients. In clinical practice, planner may provide both plans, i.e., *SE with* 6MV or *ME with* 6/15MV, for the physician to decide. Both plans have to be manually generated and therefore it essentially doubles the workload. A lack of energy selection guidance adds to the workload for treatment planners and thus impedes the clinical workflow. To build an automated energy selection tool, prior clinical plans were used to build a binary decision model (a choice of single or mixed energy) and classify the query case for the energy selection. DRRs were generated in each beam direction and the gray level histogram within the irradiation volume was calculated for each of 20 training cases. Principal component analysis (PCA) was then performed on the gray level histogram of each case (two beams combined) to reduce the data dimension and the first two component scores were used to represent each case for classification. The energy decision boundary was then determined in the 2D (first two components) feature space. Cases falling into one side of the boundary were recommended for one energy choice and cases on the other side were recommended otherwise. To evaluate the model performance, the DRR gray level histogram for the query patient was decomposed via PCA and subsequently the first two components were employed to make an energy recommendation.

#### Anatomy Shape Driven Fluence Estimation

The second step of the workflow is to generate a fluence map to achieve the optimal dose distribution within the 3D target volume. The fluence map was generated by predicting the fluence intensity of each pixel on the fluence map. Ideally, the optimal dose distribution should cover the entire breast target with prescription dose while minimizing the hot spot volume (105% of prescription dose) within the breast target. Planners iteratively refine the fluence intensity (reduce, add and smooth fluence) manually to achieve such goal. Alternatively, Eclipse^TM^ offers a module to generate the electronic compensator (“irregular surface compensator”) for each beam individually. It generates uniform dose distribution at a specified depth (e.g., 40% depth). However, since the fluence map is generated individually for each beam to achieve uniform dose at a certain 2D penetration depth plane and does not take into consideration the 3D dose distribution, dose heterogeneity often arises. A machine learning algorithm was developed to tackle this issue by learning the correlation between anatomical features and the optimal fluence map. We utilized the random forest (RF) model to summarize the relationship between input features (shape based features, including gray level intensity, penetration depth in breast target, penetration depth in lung etc.) and output variables (pixel-wise fluence intensity). RF is a highly non-linear model which initializes decision trees using randomly sampled data from a training dataset and generates a prediction by averaging the output from all trees. The RF model was trained using all 20 training plans with 150 trees. For query cases, the RF model predicted fluence intensity at the pixel level and the entire fluence map served as the fluence estimation for the corresponding beam. For ME cases, the entire predicted fluence map was divided into a low energy (6X) and high energy (15X) component. Low energy and high energy beams from the same side shares same beam parameters such as gantry angle, collimator angle and jaw sizes. The ratio of low energy fluence intensity and high energy fluence intensity for each pixel on the fluence map depends on the beamlet penetration depth and this relationship was learned from 10 training ME plans.

#### Patient-Specific Fluence Fine-Tuning

The fluence map generated from the RF model inherits the plan quality from the training cases. However, the physician may have a patient-specific requirement for the target coverage or a constraint for a high-dose volume or hot spot. The proposed third step offers the physician the opportunity to interactively fine-tune the 3D dose distribution.

The fluence fine-tuning is based upon physical principles. We aimed to specify the dose to be delivered to dose anchor points while balancing dose contribution from both beams. Dose anchor points were identified in two steps. First, they were initially identified on the iso-plane in the irradiated volume and later adjusted during the centrality correction step. Then, the centrality correction step actively balanced the beamlet penetration depth inside the breast tissue from either side for each dose anchor point. Geometric and dosimetric parameters (penetration depth, dose at anchor point etc.) of these dose anchor points were summarized from training plans to serve as baseline values and these parameters can be further adjusted to provide specific coverage or dose reduction for any query patient.

### Auto-Plan Quality Evaluation and Evaluation Metrics

The automatic planning system was implemented in C++ to improve computation speed. The proposed system was validated by comparing plan quality metrics of auto-plans with those of manually generated clinical plans using one-sided Wilcoxon Signed-Rank test. The null hypothesis was that there was no difference in the mean value of quality metrics between the two plan groups. Parameters compared included breast target V100%, breast target V105%, V105cc, lung V10Gy, lung V20Gy, lung V95%, heart mean dose (%), and plan maximum dose (%).

The breast target was contoured by physician following Radiation Therapy Oncology Group (RTOG) breast cancer atlas for RT planning (26). The significance level was adjusted to 0.006 considering Bonferroni correction (α = 0.05/8 = 0.006). All auto-plans were generated using the same energy choice as clinical plans' to compare the plan quality excluding effect of energy choice even if the system recommended otherwise.

## Results

### Model Training and Validation

**Figure 2:**
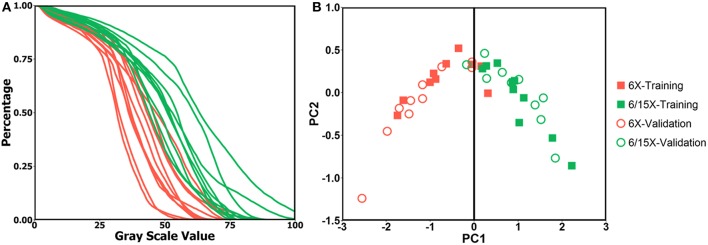
**(A)** DRR intensity histogram for single energy cases (red) and mixed energy cases (green); **(B)** PC1 and PC2 score of single energy cases (red) and mixed energy cases (green) for training dataset (solid square) and validation dataset (circle), PC1 = 0 is shown as black line.

The PCA analysis result is shown in Figure 2. The DRR intensity histogram for each patient is shown in Figure 2A. In Figure 2B, red dots represent single energy cases and green dots represent mixed energy cases. Solid squares represent training cases while circles represent validation cases. PC1 = 0 served as a good classifier with an accuracy of 19/20 for the validation cohort, meaning the model suggested the same energy combination as the clinical plans.

### Plan Quality Comparison

**Figure 3:**
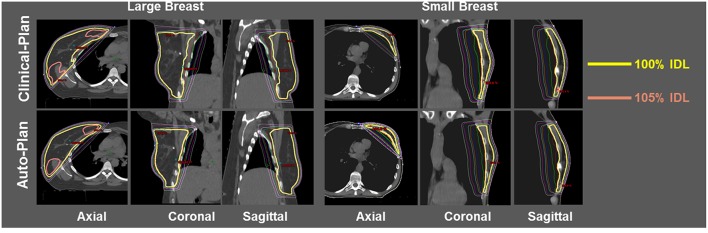
Isodose comparison between ECOMP clinical-plan (top row) and ECOMP auto-plan (bottom row) for one large breast patient (left three columns) and one small breast patient (right three columns). Yellow isodose line (IDL) denotes 100% and pink IDL denotes 105%.

Dose distribution was qualitatively compared between clinical plans generated manually and automatically generated plans. Figure 3 shows the isodose distribution comparison for one large breast case (left three columns) and one small breast case (right three columns). Overall dose homogeneity was comparable between the clinical and auto-plans. The high-dose volume (105% Rx dose volume) was similar in location as well as volume between two plan groups.

**Figure 4:**
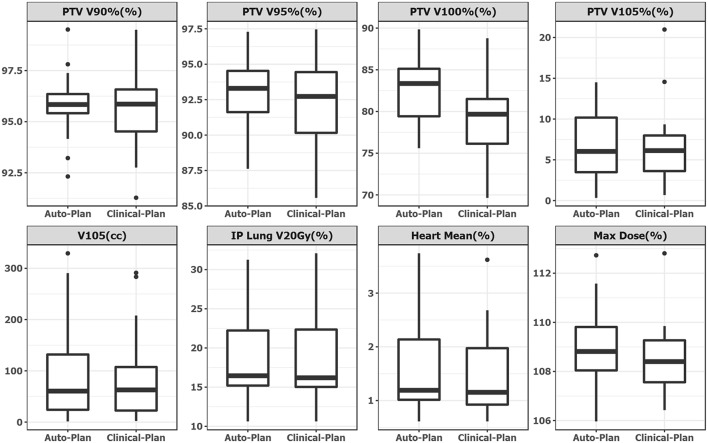
Boxplot side-by-side comparison of dose metrics between auto-plan **(Left)** and clinical-plan **(Right)**. Rectangular box denotes interquartile range. Thick line in the box denotes median. Circular dot denotes outlier data point, which is 1.5 times interquartile range above upper quartile.

**Figure 5:**
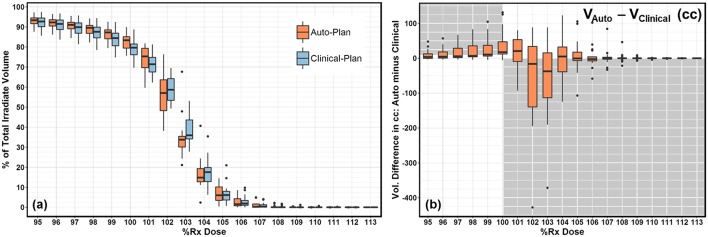
**(a)** DVH comparison between clinical-plan (blue) and auto-plan (orange). It shows breast target coverage distribution for bins in dose range of 95–113%. **(b)** DVH difference (auto-plan minus clinical-plan) in target coverage for bins in dose range of 95–113%. Positive difference in dose range of 95–100% indicates better breast target coverage. Negative difference in dose range of 100–113% indicates better hot spot volume control.

Plan quality was further compared quantitatively and summarized in Table 1. No statistical significance was observed in any dosimetric endpoints between the two plan groups except heart mean dose. This statistical significance showed systematic albeit small increase in heart mean dose, which could be caused by higher fluence near the edge around heart, or higher 15 MV beam component used for mixed energy cases. However, the overall increase was minimal and negligible (0.1%). Boxplots of dose-volume metrics are shown in Figure 4. The median and interquartile range for each endpoint were comparable between two plan groups. Figure 5 further illustrates the breast target dose-volume histograms (DVH) for each plan group. The left figure shows binned boxplots of breast target for clinical-plans (blue) and auto-plans (orange). The right figure shows the binned boxplot of breast target dose difference (auto-plan minus clinical-plan) in absolute volume. A positive difference for bins in dose range of 95–100% indicates improved coverage in the auto-plan, while a negative difference for bins in dose range of 100% and beyond indicates a reduced hot spot volume in the auto-plan and also quality improvement. The preferable area is shaded in gray (upper left and bottom right quadrant), a.k.a. the results in the gray zones indicating improvement of plan quality.

**Table 1:** Dosimetric comparison between the auto-plans and clinical-plans.

**Plan metrics**	**Auto-plan**	**Clinical-plan**	**Wilcoxon**
	**Mean (SD)**	**Mean (SD)**	**Signed-Rank**
Breast Target V90%	95.8% (1.6%)	95.5% (1.8%)	*p = 0.976*
Breast Target V95%	93.0% (2.4%)	92.3% (2.9%)	*p = 0.999*
Breast Target V100%	82.5% (4.2%)	79.3% (4.8%)	*p* > *0.999*
Breast Target V105%	6.9% (4.0%)	6.4% (4.8%)	*p = 0.314*
Breast Target V105% (cc)	95.2 (90.7)	83.9 (87.2)	*p = 0.108*
Lung V20Gy	18.9% (5.9%)	18.9% (6.1%)	*p = 0.060*
Heart Mean (%)	1.6% (0.9%)	1.5% (0.8%)	*p < 0.001*
Plan Maximum Dose (%)	109.1% (1.6%)	108.5% (1.4%)	*p = 0.057*

^*^SD, Standard Deviation. Statistical significance was tested via Wilcoxon Signed-Rank test. p < 0.006 denotes statistical significance (Bonferroni correction adjusted).

### Planning Efficiency Comparison

Total optimization time for auto-plans was <20 s. Even as a stand-alone platform, the entire process including data transferring from and to the treatment planning system can be accomplished within 5 min. This is substantially lower than the manual process which ranges from 30 min to 4 h in our clinic.

## Discussion

In this paper we described the development and validation of a fully automated whole breast treatment planning system that can generate breast radiotherapy plans with quality comparable to the current clinical standard while reducing the planning time to within minutes. This automatic planning system offers an energy selection tool, provides a fluence map estimation that is based on anatomy and fine-tunes the fluence and dose distribution based on physical principles and according to the clinical needs for each patient. Validation using clinical cases demonstrated promising applicability and quality for the proposed system. The automated workflow could substantially reduce the planning time, and therefore enable physicians and planners to very rapidly tailor/adapt breast radiotherapy for individual patient anatomical and tumor characteristics. Moreover, the automatic planning system has the potential to improve the overall quality and consistency of treatment planning across treatment centers where planner may have different levels of experience.

As reflected by the validation cohort, the dosimetric parameters between auto-plans and manually generated clinical-plans were overall comparable. No statistically significant difference (except heart mean dose) was observed indicating comparable quality between the two plan groups. More importantly, the automated workflow was able to achieve similar 105% Rx hot spot volume, which may take hours for a human planner to achieve. The system is able to prepare a tangential beam plan within a very short period of time, and therefore multiple plans (with tradeoffs) can be generated simultaneously to reflect different clinical preferences, such as improved coverage, reduced hot spot or reduced lung dose etc. Such flexibility is an important step toward implementing personalized treatment for each individual patient. In addition, the proposed system does not impose any requirement on the dose calculation algorithm. The fluence fine-tuning relies on the current dose parameters of the patient, which can be realized with one-iteration of dose calculation using the RF model fluence estimation. Finally, the system does not require re-training the model for different institutions, as the fluence estimation model is primarily based on patient characteristics.

The proposed automatic treatment planning solution for WBRT is presented with cases in the Eclipse TPS. It's worth mentioning that the system is transferrable between TPSs from different vendors. Computed tomography (CT) and structure set based on the DICOM compatible format are required as the input to generate initial shape driven fluence estimation. Admittedly the dose calculation across different vendors could bear difference, however we have already taken this variation into consideration in the subsequent fluence fine tuning step. This proposed framework offers the advantage of independence on TPS or dose calculation model, which minimizes the effort needed for distribution or TPS upgrade. A future study is warranted in this regard to evaluate the inter-treatment-planning-system performance.

The proposed automatic WBRT treatment planning solution provides substantial reduction in the treatment planning time for a busy clinic. It would allow dosimetrists to focus on more complicated tasks. Although the plan can be automatically generated, we still aim to have dosimetrist to review and finalize the plan before presenting it for final approval. Dosimetrist can make edits if necessary to tailor the plan for a specific patient. In order to facilitate this process, a following study is underway to offer more flexibility based on the planner's need, such as boosting tumor bed coverage etc.

There are several limitations of this work. First, as mentioned before, more flexible choices could be provided to the planner to customize the isodose distribution for a specific patient. The current format of the model reflects a somewhat averaged plan quality from the training population. Second, no special consideration has been given to the tumor bed. For shallower located tumor bed, more skin dose is needed to fully cover the entire tumor bed. This feature can be implemented in the next version of the solution. Third, this work focuses on automating the most time-consuming step in the treatment planning process, namely fluence editing. In the future study, we would focus on providing a closed-loop solution for breast cancer radiation therapy treatment planning starting from automating structure contouring and beam setup, which would further minimize human effort.

WBRT using ECOMP tangential fields has the advantage of uniform dose distribution across the 3D volume over other treatment modalities such as FiF. Due to the lengthy treatment planning process and other logistical issues, ECOMP is currently not the most popular choice for WBRT. Conventionally, 3D treatment using physical wedges were utilized, but gradually this was replaced by FiF, IMRT, and VMAT to achieve more optimal dose homogeneity. Automating ECOMP planning using tangential fields would offer a more efficient way of providing optimized plans and could foreseeably reduce the workload of medical dosimetrist and allow them to focus on more complicated cases. In an era when society calls for value-based care, this approach is a planning-efficient, low toxicity and inexpensive technique.

## Conclusion

With the hope of improving the whole breast radiation therapy treatment planning workflow and more importantly providing better patient care, we proposed an automatic treatment planning solution utilizing anatomy and physics rules for the tangential style ECOMP whole breast radiotherapy. Auto-plans provided similar target volume coverage and hot spot volume reduction as compared to manually generated clinical-plans. The automated process reduces treatment planning time from 30 min−4 h to <5 min. Its high efficiency and near-real-time fine-tuning allows physicians to spend less time waiting for plans, and to focus more on providing evidence-based, personalized care to breast cancer patients.

## Data Availability

All datasets generated for this study are included in the manuscript and/or the supplementary files.

## Ethics Statement

This study has been approved by DUHS Institutional Review Board under Amendment ID Amd002_Pro00034599.

## Author Contributions

YS and TL performed the model development and treatment planning comparison. SY, RB, and JKH reviewed the plan and provided the feedback for model improvement. FFY and YG reviewed the experiment design, paper content, and statistical analysis. JW supervised the entire study and revised this paper.

### Conflict of Interest Statement

YS, TL, FFY, YG, and JW have filed International Patent Application No. PCT/US2016/063204 for this work licensed to Duke University. The remaining authors declare that the research was conducted in the absence of any commercial or financial relationships that could be construed as a potential conflict of interest.
